# Cytokines assets in PLWH in two-drug dolutergravir based or three-drug antiretroviral regimen

**DOI:** 10.1186/s12879-024-09565-w

**Published:** 2024-07-03

**Authors:** Katia Falasca, Claudio Ucciferri, Alessandro Di Gasbarro, Paola Borrelli, Marta Di Nicola, Carla Frisenda, Erica Costantini, Lisa Aielli, Marcella Reale, Jacopo Vecchiet

**Affiliations:** 1 Clinic of Infectious Diseases, Department of Medicine and Science of Aging, University “G. d’Annunzio”, Via dei Vestini, Chieti, 66100 Italy; 2 Laboratory of Biostatistics, Department of Medical, Oral and Biotechnological Sciences, University “G. D’Annunzio”, Via dei Vestini, Chieti, 66100 Italy; 3 Department of Innovative Technologies in Medicine and Dentistry, University “G. d’Annunzio”, Via dei Vestini, Chieti, 66100 Italy

**Keywords:** HIV, DTG, 2DRs, 3DR, Inflammation

## Abstract

To minimize the toxicity and impact of combined antiretroviral therapy (cART) on the lifestyle of people living with Human Immunodeficiency Virus (PLWH), scientific community evaluated the efficacy, safety and sustained virologic response of two drugs antiretroviral regimens, in particular dolutegravir (DTG). The effects of deintensification therapy on inflammatory settings are currently unknown in PLWH. Thus, our study explored the inflammatory state in virologically suppressed HIV individuals between patients in treatment with a DTG-containing dual therapy (2DR) versus triple regimen therapies (3DR). We enrolled a total of 116 subjects in *2DRs* or *3DRs* regimens, and the plasma levels of pro- and anti-inflammatory cytokines (in particular IL-1β, IL-10, IL-18, IL-33, IL-36 and IFN-γ) have been evaluated. CD4 + cell’s median value was 729.0 cell/µL in the 3DR group and 771.5 cell/µL in 2DR group; the viral load was negative in all patients. Significant differences were found in levels of IL-18 (648.8 cell/µL in 3DR group vs. 475.0 cell/µL in 2DR group, *p* = 0.034) and IL-36 (281.7 cell/µL in 3DR group vs. 247.0 cell/µL in 2DR group, *p* = 0.050), and a correlation between IL-18 and IL-36 was found in 3DR group (rho = 0.266, *p* = 0.015). This single-center retrospective pharmacological study confirms the absence of significant differences in IL-1β, IL-10, IL-33, and IFN-γ levels between patients on two-drug antiretroviral regimens compared to patients on 3DR antiretroviral regimens. Patients in 2DR show greater control over IL-18 and IL-36 serum levels, cytokines related to an increased cardiovascular risk and development of age-related chronic diseases. Based on our results, we suggest that DTG-based 2DR antiretroviral regimens could be associated with better control of the chronic inflammation that characterizes the population living with HIV in effective ART.

## Introduction

The introduction of combined antiretroviral therapy (cART) significantly decreased the HIV-related morbidity and mortality among people living with HIV, and their life expectancy gradually became comparable to the general population [1]. With the increase in life expectancy among PLWH, the number of people over 50 years of age increases [2] which leads to an increase in age-related comorbidities. [3]. A plethora of mechanisms contribute to the pathogenesis of non-HIV-related diseases in HIV patients [3], and inflammation plays a central role [4]. Despite cART related viral suppression, inflammatory activation persists [5] inducing a proinflammatory and procoagulant condition [6–8], resulting in increased morbidity and mortality. The Strategies for Management of Antiretroviral Therapy (SMART) trial demonstrated the association between IL-6, D-dimer and persistent immune system activation underlying the onset of non-AIDS-related morbidity and mortality in PLWH receiving effective treatment [9]. Although the mechanisms underlying this phenomena are not yet fully known, the HIV reservoirs [10], microbial translocation [11], antiretroviral therapy, [12] were considered. To minimize the impact of cART, not only on PLWHs’ lifestyle but also on a drug toxicity, the efficacy, safety and virologic performance (SVR) of antiretroviral dolutegravir (DTG) based regimens containing only two drugs [13] have been evaluated and appear to be a good therapeutic option [14, 15]. Proinflammatory cytokines of IL-1 superfamily, including IL-1β, IL-18, IL-33 and IL-36 showed several structural similarities and common signaling pathway which resulted in the activation of NF-κB and MAPK and then promoting the transcription of several inflammatory genes. The immunosuppressive cytokine IL-10, notoriously, was correlated with viral load and was reduced after successful antiretroviral therapy. Since the effects of therapeutic deintensification on inflammatory status, currently, are not well known in HIV. In this study, we compared the inflammatory state in virologically suppressed PLWH in DTG-based dual therapy (2DR) and patients with different triple therapy (3DR).

## Materials and methods

### Study design and patients

This was a cross sectional study conducted on outpatients who were being followed up at the Infectious Diseases Clinic, University “Gabriele d’Annunzio” SS. Annunziata in Chieti between 01 January 2022 and 01 March 2022.

We included patients who met all the following criteria: age > 18 years, HIV-1 infection for at least 60 months under stable (without any pharmacological therapy change over the last 48 weeks) and effective (HIV RNA undetectable in last 2 blood samples) cART.

The exclusion criteria were: any acute infection at enrollment or in last three months, non-steroidal anti-inflammatory drug (NSAID) therapy in the last seven days before blood sampling, concomitant therapy with corticosteroids, current pregnancy, cardiac ischemia or stroke in last 6 months.

### Plasma samples

To avoid a day-to-day variability in cytokine release determined by eating and physical activity, venous blood samples were collected using venous blood drawn from the antecubital vein, in the morning between 08:00 and 9.00 h.after overnight fasting, and the following biomarkers were measured: blood count with leukocyte formula, C-reactive protein (CRP), creatinine, blood urea nitrogen (BUN), eGFR, aspartate amino transferase (AST), alanine amino transferase (ALT), gamma glutamyl transpeptidase (γGT), alkaline phosphatase, dehydrogenated lactate (LDH), total cholesterol, LDL-cholesterol, HDL-cholesterol, triglycerides, cystatin-C, sodium, potassium, uric acid, 25-OH-vitamin D; plasma cystatin C was determined using the BN II system with the nephelometric technique. (BN II System - Siemens Healthcare Diagnostic, Inc).

CD4 + and CD8 + T cell counts were obtained by flow cytometry of lymphocyte subpopulations. Plasma viral load (HIV-RNA) was determined using the “Amplicor” method (Roche Molecular Diagnostics, Milan, Italy) with a detection limit of > 27 HIV RNA copies/mL of plasma.

All laboratory tests were performed at the Clinical Pathology of the University ‘G. D’Annunzio’ SS Annunziata Hospital of Chieti.

### Cytokines levels

The surplus of blood contained in plasma tubes was centrifuged at 4000 rpm for 5 min, then the plasma thus obtained was stored in 2 mL containers, cataloged and frozen at -20 °C until the analysis of cytokine levels, carried out within 90 days of storage. We have evaluated plasma concentration of proinflammatory Interleukins (IL)-1β, IL-10, IL-18, IL-33, IL-36, and of the anti-inflammatory cytokine IL10, and of Interferon (IFN)-γ cby enzyme-linked immunosorbent assays (ELISA) using commercially available kits (Diaclone SAS; France; Cusabio, Houston, USA and Boster, CA, USA). All samples for a given assay, including standards, were analyzed in duplicate at the same time following the manufacturer’s instructions. The lower detection limit of assay was ≤ 1 pg/mL for IL-18, ≤ 4,69 pg/mL for IL-1β, ≤ 4,9 pg/mL for IL-10, ≤ 5 pg/mL for IFNγ, ≤ 12,2 9 pg/mL for IL-33, ≤ 19,5 9 pg/mL for IL-36. Standards and samples were analyzed in duplicate. Cytokine levels were calculated plotting the optical density (O.D.) of each sample against the standard curve. Values that differed from the mean of duplicate by greater than 10% were not considered for further analysis. The variation coefficient of both interassay and intra-assay was < 5%.

### Statistical analysis

Normality distribution for quantitative variables was assessed by the Shapiro-Wilk. Descriptive analysis was carried out using median and interquartile range (IQR) for the quantitative variables and percentage values for the qualitative ones. Test. Pearson’s chi-square test or Fisher’s exact test was used to evaluate the association between categorical variables while the non-parametric Wilcoxon rank-sum test for unpaired two-samples to evaluate the differences between continuous variables and outcome considered.

The relationships among cytokines were tested using Spearman’s correlation coefficient (rho) in 3DR group and 2DR group Statistical significance was set at the level of ≤ 0.05, All analyses were performed using Stata software v17.1 (StataCorp, College Station, USA).

## Results

### Descriptive analysis of the sample

A total of 116 patients were enrolled: 89 (76.7%) were male and the median age was 51 years (IQR 43.0–59.0). The median years since the diagnosis of HIV infection was 15 years (IQR 8.5–24.0). Forty six patients (39.7%) were smokers, 44 (38.3%) had elevated blood pressure values, 6 (5.2%) had type II diabetes mellitus, depressive syndrome was present in 41 (35.3%) patients, lipodystrophy in 7 (6.0%), dyslipidemia in 83 (71.6%) and chronic renal failure in 12 (10.4%). Body Mass Index (BMI) at the time of study had a median of 25.6 kg/m^2^ (IQR 23.3–27.9).

The risk factors for HIV infection were: unprotected homosexual sex in 41 (35.3%), unprotected heterosexual sex in 55 (47.4%), promiscuous use of syringes for intravenous drug intake in 17 (14.7%), unprotected bisexual sex in 2 (1.7%) and vertical transmission in 1 subject (0.9%).

During the study, 82 patients were in cART with 3 antiretroviral drugs (3DR group) and 34 patients in DTG-based cART composed of two molecules (2DR group). The comparison analysis did not reveal statistically significant differences between groups for socio-demographic characteristics, specifically for gender (male 75.6% in 3DR group vs. 79.4% in 2DR group, *p* = 0.659) and median age (51.0 (IQR 43.0–60.0) in 3DR group vs. 50.5 (IQR 44.0–57.0) in 2DR group, *p* = 0.673 ).

CD4 + T cells median value was 729.0 cell/uL (IQR 492.0-934.0) in 3DR group and 771.5 (IQR 589.0-956.0) in 2DR group, CD8 + T cells were a median of 729.5 cell/uL (IQR 550.5–993.0) in 3DR group and a median of 737.5 cell/uL (IQR 603.0-1021.0) in 2DR group, CD4/CD8 ratio has a median value of 0.9 (IQR 0.7–1.3) in 3DR group and a median value of 1 (IQR 0.7–1.5) in 2DR group. The viral load was undetectable in all patients.

### Analysis of hematochemical and viro-immunological parameters

The main hematochemical values measured in the sample during the study, were within normal ranges.Significant differences emerged in ALT [a median of 22.5U/L (IQR 16.0–32.0)] in 3DR group vs. a median of [16 U/L (IQR 14.0–21.0) in 2DR group, *p* = 0.011] and γGT levels [a median of 34 U/L (IQR 21.0–54.0)] in 3DR group vs. a median of 22.5 [(IQR 16.0–38.0) in 2DR group, *p* = 0.018)]: despite the statistical significance revealed, the median values fall within the reference interval. No other significant differences in biochemical parameters were found (Table 1).

**Table 1:** Hematology and biochemical parameters in 3DR and 2DR groups

	3DR (*n* = 82)	2DR (*n* = 34)	*P*-value
RBC (cells/mmc)	4665000.0 (4360000.0-5050000.0)	4590000.0 (4260666.0-4980000.0)	0.817
Hemoglobin (g/dl)	14.6 (13.7–15.3)	15.1 (14.2–15.9)	0.077
Platelets (cells/mmc)	201500.0 (175000.0-257000.0)	221000.0 (196000.0-252000.0)	0.205
WBC (cells/mmc)	6405.0 (5290.0-7740.0)	7025.0 (5980.0-7440.0)	0.378
Neutrophils (cells/mmc)	3310.0 (2570.0-4570.0)	3345.0 (2710.0-4520.0)	0.636
Lymphocytes (cells/mmc)	2090.0 (1690.0-2620.0)	2150.0 (1900.0-3000.0)	0.360
Monocytes (cells/mmc)	530.0 (430.0-620.0)	555.0 (410.0-670.0)	0.524
Eosinophils (cells/mmc)	165.0 (100.0-210.0)	140.0 (110.0-220.0)	0.897
Basophils (cells/mmc)	40.0 (30.0–60.0)	40.0 (30.0–50.0)	0.949
Creatinine (mg/dl)	1.0 (0.8–1.1)	1.0 (0.9–1.2)	0.098
eGFR (ml/min/1,7 m2)	80.9 (71.9-101.5)	77.8 (72.0–89.0)	0.119
Total Cholesterol (mg/dl)	187.0 (161.0-213.0)	184.5 (171.0-198.0)	0.791
LDL-Cholesterol (mg/dl)	126.0 (101.0-152.0)	127.0 (115.0-138.0)	0.704
Triglycerides (mg/dl)	107.5 (72.0-171.0)	100.0 (73.0-140.0)	0.507
Uric acid (mg/dl)	5.5 (4.6–6.2)	5.5 (4.2–6.6)	0.938
AST (U/L)	20.0 (17.0–25.0)	18.0 (16.0–25.0)	0.247
ALT (U/L)	22.5 (16.0–32.0)	16.0 (14.0–21.0)	**0.011**
GGT (U/L)	34.0 (21.0–54.0)	22.5 (16.0–38.0)	**0.018**
Alkaline phosphatase (U/L)	66.5 (51.0–83.0)	62.0 (54.0–73.0)	0.381
LDH (U/L)	195.0 (176.0-220.0)	192.0 (171.0-215.0)	0.609
Vitamin D (ng/ml)	31.5 (23.2–36.1)	30.4 (24.8–44.4)	0.339
CRP (mg/L)	2.4 (1.0-5.5)	1.2 (0.7–3.2)	0.052
Cistatina C (mg/L)	0.9 (0.8-1.0)	0.9 (0.8–0.9)	0.407
HIV Infection			
AIDS events	12 (14.7%)	3 (8.9%)	0.548
HIV duration	15.5 (9–24)	12.5 (7–24)	0.291
cART			
INI	20 (12.2%)	0 (0.0%)	-
PI	26 (15.8%)	0 (0.0%)	
NNRTI	36 (21.9%)	0 (0.0%)	
TAF/FTC	64 (39.1%)	0 (0.0%)	
ABC/3TC	18 (10.0%)	0 (0.0%)	
DTG/3TC	0 (0.0%)	23 (67.7%)	
DTG/RPV	0 (0.0%)	11 (32.3%)	
cART duration	12.0 (8.0–17.0)	12.0 (7.0–16.0)	0.530
Nadir CD4+(cells/mmc)			
>500	14.0 (17.1%)	6 (17.7%)	0.957
200–500	41 (50.0%)	16 (47.1%)	
<200	27 (32.9%)	12 (35.3%)	

Data are expressed as median and interquartile range (IQR)

*p-values are for Wilcoxon rank-sum test or Pearson chi-square test

RBC = Red blood cells; WBC = White blood cells; eGFR: Estimated Glomerular Filtration Rate, AST: aspartate transaminase, ALT: alanine transaminase; GGT: GammaGT; LDH: Lactate dehydrogenase; PCR: C-reactive protein; INI: Integrase inhibitors; PI: Protease inhibitors; NNRTI: Non-nucleoside reverse transcriptase inhibitors

**Fig. 1:**
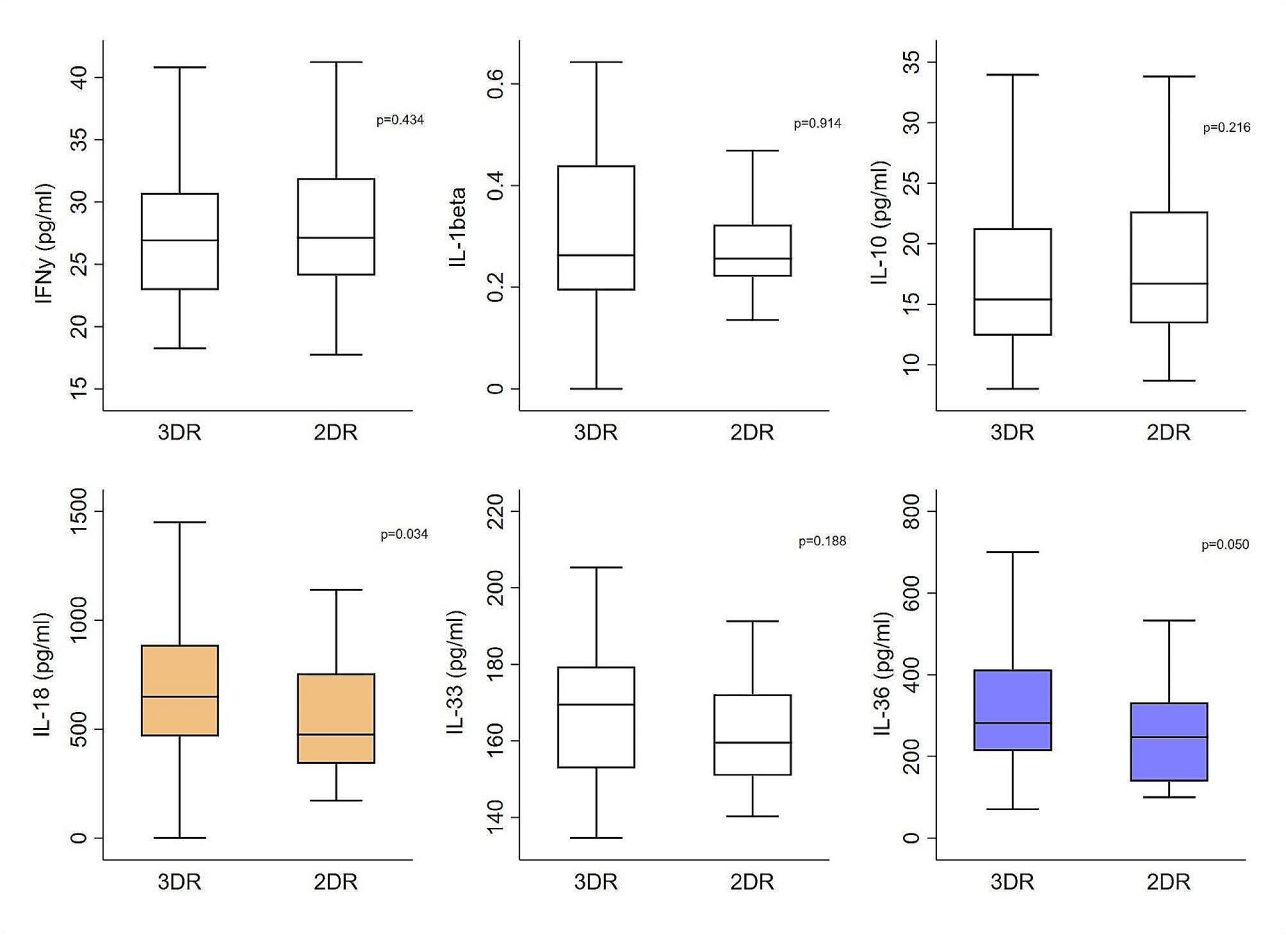
Box plots show the median levels of the cytokines in the two comparison groups. *P*-values are for Wilcoxon rank-sum test

Serum levels of IFNγ levels were 26.9 pg/ml (IQR 22.9–30.7) in 3DR group and of 27.1 pg/ml (IQR 24.1–31.9) in 2DR group. The IL-10 was 15.4 pg/dl (IQR 12.4–21.3) in the 3DR and of 16.7 pg/dl (IQR 13.4–22.7) in the 2DR; the IL-33 and IL-1β plasma levels were respectively 169.5 pg/ml (IQR 152.8-179.5) and 0.3 (IQR 0.2–0.4) in 3DR group, and 159.5 pg/ml (IQR 150.9-172.2) and 0.3 pg/dl (IQR 0.2–0.3) in 2DR group. Significant differences were found between the two groups in the levels of IL-18 (648.8 pg/ml in 3DR group vs. 475.0 pg/ml in 2DR group, *p* = 0.034) and IL-36 (281.7 pg/ml in 3DR group vs. 247.0 pg/ml in 2DR group, *p* = 0.050) (Fig. 1). For these cytokines found to be significant in comparison, the correlation between them in the two groups was calculated separately.Spearman rank correlation showed a positive correlation between IL-18 and IL-36 serum levels in 3DR group (rho = 0.266, *p* = 0.015).

## Discussion

This study aims to evaluate plasma levels of pro- and anti-inflammatory cytokines in PLWH in 2DR and 3DR cART. Our results show that the anti-inflammatory cytokines profile measured in this study was not significantly different, while the pro-inflammatory cytokines IL-18 and IL-36 were significantly higher in PLWH receiving a 3DR anti-HIV therapy compared to those receiving a 2DR therapy.

IL-18 is a member of the IL-1 family of cytokines, produced constitutively by macrophages, endothelial cells, vascular smooth muscle cells and dendritic cells: this molecule has a potent proinflammatory activity that promotes the release of other cytokines, chemokines and cell adhesion molecules. High levels of IL-18 have been found in patients with metabolic syndrome [16] and have been correlated with the development of atherosclerotic plaques, plaque instability and acute cardiovascular events [17, 18], with cardiovascular risk score (PROCAM, DAD, FRS, ASCVD) [19] and can become predictive of coronary events [20].

IL-36 has pro-inflammatory effects, and is expressed in numerous cells such as T cells, monocytes/macrophages, dendritic cells (DCs), keratinocytes, Langerhans cells, lung and gut cells. IL-36 plays an important role in immune cell activation, antigen presentation and inflammatory diseases [21–23], showing stimulatory effects on DCs and T-cells and interfacing innate and adaptive immune responses in viral, bacterial and fungal infections [24]. Recently, IL-36 has aroused great interest because of its dysregulation in inflammatory diseases, in fact the serum levels of IL-36 were found to be significantly higher with coronary artery disease, correlated with TNF-α, IL-6 and IL-32 levels and coronary artery stenosis [25]. In ApoE knockout mice the IL-36γ can exert atherosclerosis-promoting effects by augmenting macrophage foam cell formation and uptake of oxidized low-density lipoproteins [26]. IL-36 signaling also induces the release of profibrotic mediators, suggesting a role in fibrotic disorders affecting kidneys, lung, and intestines [27]. To date, IL-36’s involvement in PLWH is unknown, and in this study we showed that PLWH in 2DR regimens have significantly lower IL-18 and IL-36 levels than their counterparts in 3DR, potentially reducing the cardiovascular risk, the positive correlation between IL-18 and IL-36 found in the 3DR group shows a strong association with cardiovascular risk.

The levels of IL-1β and IL-33, cytokines with important roles in metabolic homeostasis, viral infection, inflammation and carcinogenesis, are not statistically significant different in the two groups, as well as the levels of the immunoregulatory IL-10 and immunostimulatory IFN-γ.

Lower levels of IL-18 and IL-36 in the 2DR group could indicate a good control on the inflammatory state even in cART regimens composed by only two drugs. Thus, DTG-based cART dual therapies acting on inflammation state might be able to reduce, over time, the cardiovascular risk of the HIV patient which, nowadays, remains higher than the general population [28–30]. This study has limitations, particularly due to the small sample size. As a result, the tests conducted can only be attributed to the specific comparisons made and have not allowed for the development of predictive models correlating the results with levels of inflammatory markers. Conducting future studies with a larger population will enable us to explore this relationship further.

## Conclusion

This single-center retrospective pharmacological study confirms the absence of significant differences in IL-1β, IL-10, IL-33, and IFN-γ levels between patients on dolutegravir based two-drug antiretroviral regimens compared to patients on three-drug antiretroviral regimens.

Interestingly, the patients in dual therapies show greater control over IL-18 and IL-36 serum levels, cytokines related to an increased cardiovascular risk and chronic diseases related to ageing.

These data demonstrating that regimens featuring two antiretroviral agents, DTG based, could be associated with a good control on chronic inflammatory state in PLWHs in cART, could be a further incentive for the clinician to approach the pharmacological simplifications of HIV regimen.

## Data Availability

The datasets used and/or analyzed during the current study are available from the corresponding author on reasonable request.
